# Recent advances in understanding and managing sepsis

**DOI:** 10.12688/f1000research.15758.1

**Published:** 2018-09-28

**Authors:** Daniela Berg, Herwig Gerlach

**Affiliations:** 1 Department of Anesthesia, Critical Care Medicine, and Pain Management, Vivantes – Klinikum Neukoelln, Berlin, Berlin, Germany

**Keywords:** Sepsis, Sepsis Pathophysiology, Sepsis Definition, Sepsis Management

## Abstract

The last two to three years provided several “big steps” regarding our understanding and management of sepsis. The increasing insight into pathomechanisms of post-infectious defense led to some new models of host response. Besides hyper-, hypo-, and anti-inflammation as the traditional approaches to sepsis pathophysiology, tolerance and resilience were described as natural ways that organisms react to microbes. In parallel, huge data analyses confirmed these research insights with a new way to define sepsis and septic shock (called “Sepsis-3”), which led to discussions within the scientific community. In addition to these advances in understanding and defining the disease, follow-up protocols of the initial “sepsis bundles” from the Surviving Sepsis Campaign were created; some of them were part of quality management studies by clinicians, and some were in the form of mandatory procedures. As a result, new “bundles” were initiated with the goal of enabling standardized management of sepsis and septic shock, especially in the very early phase. This short commentary provides a brief overview of these two major fields as recent hallmarks of sepsis research.

## Introduction

Currently, sepsis and septic shock with subsequent multi-organ failure are the leading causes of death in adult intensive care units (ICUs). Although surgical and pharmacological approaches in sepsis therapy are continually improving, epidemiological studies show an increased incidence of sepsis over the last 20 years ^1^. In the past few decades, the high prevalence of sepsis and its high economic impact have led to the development of several projects intended to allow better recognition and more accurate description of the course of the disease ^2^.

Sepsis is one of the oldest described illnesses. The term sepsis is derived from the ancient Greek term “σῆψις” (“make rotten”) and was used by Hippocrates around 400 BCE to describe the natural process through which meat decays and swamps release decomposing gases but also through which infected wounds become purulent ^3^. After this recognition, it took over 2,000 years to establish the hypothesis that it is not the pathogen itself but rather the host response that is responsible for the symptoms seen in sepsis ^4^.

In the last 40 years, one major field of sepsis research was the basic cellular and molecular biology to understand the exact mechanisms behind why the body sometimes reacts with an overwhelming inflammation to infections but sometimes does not. Recent research will be described in the first part of the following text, which gives a possible response to this question.

Clear definitions are of great importance in the medical field, as appropriate treatment of illness demands a correct preceding diagnosis. This is not always simple, and, particularly in emergency and intensive care medicine, fast and reliable diagnosis is needed to treat acute illness. The challenge of fast diagnosis of sepsis is that this syndrome is based on highly complex pathophysiological pathways that may show varying clinical signs and symptoms. Therefore, a brief review of former and new definitions of sepsis will follow; it should be interpreted in the context of the newly described approaches to pathophysiology.

Fast detection and initial treatment of sepsis are of utmost importance. Since 2004, the Surviving Sepsis Campaign (SSC) has developed several guidelines for the management of sepsis and septic shock. From 2005 to 2010, “sepsis bundles” were tested to demonstrate that a protocolized approach in the initial phase of the disease leads to a better outcome. Since this large trial, several similar approaches have been published, and recent articles confirmed the importance of time until treatment as a prognostic factor for patients. These studies as well as the newly described bundles are part of the second section of this brief review.

## Old and new approaches to understand the disease “sepsis”

The synonym of sepsis, “blood poisoning”, which has been used for centuries and is still popular among the non-medical population, is an inadequate term for intensive care specialists. A teleological definition was proposed by Hugo Schottmüller in 1914: “Sepsis is present if a focus has developed from which pathogenic bacteria, constantly or periodically, invade the blood stream in such a way that this causes subjective and objective symptoms” ^5^. This definition is problematic and increasingly being dismissed, as it is based on subjective clinical observations. In addition, it insinuates an incorrect pathophysiological rationale, as it assumes that bacteria themselves spread. However, today one assumes that the body produces its own transmitters as a response to the infection and that these spread systemically, thus affecting peripheral organs ^4^.

In local infections, a normal inflammatory host response controls the focus; a dysregulation of the host response, however, leads to macrocirculatory and microcirculatory failure, by which single or multiple organ failure is induced ^6^. The lungs, kidneys, and cardiovascular system are the most affected organs during sepsis and septic shock ^7^. This, however, is based on clinical assessment of organ function by routine biomarkers and might not withstand a thorough check by modern cell biology tools. Furthermore, it still does not give an answer to the key question of why some patients (for instance, in cases of severe pneumonia or meningitis due to *Streptococcus pneumoniae*) react excessively in terms of hyper-inflammation and “cytokine storm” (see below) whereas others have no symptoms although the same microbe can be detected on their skin or upper airways ^8^. The “old” theory is that the latter persons are simply “resistant” (that is, their inflammatory response keeps the contamination under control). However, there are two phenomena which contradict this theory. First, if carriers without symptoms have such a strong resistance, why are they still carriers? Second, if these persons have such a successful “inflammatory response”, why is it not possible to demonstrate this with serum biomarkers?

In a landmark article, Weis *et al.* describe a biological pathway for how this may be declared ^9^: it was found that blood glucose levels influence the mechanisms of “tolerance” against infections. “Tolerance” (or “resilience”) is a form of “defense strategy against infection that preserves host homeostasis without exerting a direct negative impact on pathogens” ^9^. In other words, the host organism coexists with the microbes; of course, this may change if this tolerance is disturbed (“dysregulated”) by, for example, other infections, pregnancy, splenectomy (in case of *Streptococcus pneumoniae*), or older age. Interestingly, in many of those cases where this disease tolerance fails, the clinical symptoms of sepsis often exert much more dramatic courses than “classical” infections. These recent findings about dysregulation in the pathogenesis of sepsis perfectly fit the new definition (see next section). Finally, the option to differentiate between the individual “type of host response” may foster research to enable the practice of more personalized medicine in patients with sepsis as was suggested for other life-threatening diseases such as acute respiratory distress syndrome ^10^.

## From understanding to defining sepsis

As already mentioned, the former understanding of sepsis as a hyper-inflammatory response to infection, often accompanied by a fast “cytokine storm” ^11^, was the basis for former sepsis definitions: the US critical care specialist Roger Bone organized a consensus conference in 1992 and suggested that the sepsis definition include the aspect of host response. Here, the term systemic inflammatory response syndrome (SIRS), which is still commonly used, was defined ^12^. If SIRS occurs without infection (for example, in the case of burns and pancreatitis and in the post-operative setting), the condition is defined only as SIRS; similarly, an infection without SIRS does not equal sepsis. Only when the two criteria are seen in combination can the diagnosis of sepsis be made. Therefore, sepsis was defined as “a systemic inflammatory response syndrome to infection” that may be seen when two or more of the following criteria are fulfilled: heart rate of more than 90 beats/minute, core temperature of more than 38°C or less than 36°C, respiratory rate of more than 20 breaths/minute or partial pressure of carbon dioxide (PaCO _2_) of less than 32 mmHg, and white blood cell count of more than 12,000/mL or less than 4,000/mL or more than 10% immature neutrophils.

This definition of sepsis (“Sepsis-1”) is still most commonly used. Merely 10 years after the consensus conference hosted by Bone *et al*. ^12^, several experts met in Washington, D.C., to discuss a new definition of sepsis. The experts reached the following conclusions:

1. The current concepts of sepsis and septic shock seem, in principle, useful for clinical routine and research.
2. These definitions, however, do not allow precise staging of patients or prediction of host response and infection.
3. Although SIRS is a useful approach, criteria are too sensitive and non-specific.
4. An elaborate list of signs and symptoms of sepsis would better reflect the clinical response to a systemic infection.
5. A hypothetical model should be developed that better stages sepsis, better characterizes sepsis on the basis of predisposing factors and comorbidities, better reflects the type of original infection, better describes the host response, and better quantifies the extent of resulting organ dysfunction.

In this manner, a classification system allowing the stratification of patients with sepsis, “PIRO” (today called “Sepsis-2”), was developed at this conference in 2001 ^13^. “P” stands for predisposition, “I” for type and extent of the primary insult (in the case of sepsis, primary infection), “R” for type and extent of host response, and “O” for extent of organ dysfunction. The benefit of the PIRO model is that it enabled one to separate morbidity due to the infection itself from secondary morbidity that develops through the host response. The introductions of a PIRO model, however, remained theoretical, even though there have been several attempts to introduce a point system that enables scoring of patients with sepsis ^14^.

In a roughly two-year process with extended and complex biometric evaluations, a new approach (“Sepsis-3”) was developed that is based on patient data from several validated sources and that was published in the form of three articles in 2016 ^15– 17^. What is new in this concept? On the one hand, it is the omission of SIRS as a factor in the definition. The new Sepsis-3 defines sepsis as “a life-threatening organ dysfunction caused by a dysregulated host response to infection” ^15^. Therefore, if no organ dysfunction is seen, one may speak only of an infection, not of “sepsis”. The term “severe sepsis” is superfluous, as its criteria (organ dysfunction) are already included in the new definition of sepsis. The term “septic shock” remains; however, it now also includes an elevated lactate level of more than 2 mmol/L as an additional factor.

Part of the new Sepsis-3 definitions is SOFA as a grading score for defining acute organ dysfunction (“Sequential [Sepsis-related] Organ Failure Assessment Score”, or SOFA score) ^18^. This score allocates points according to pathological change in six different organ systems; an increase in the total SOFA score by at least two points (with negative patient history, a score of 0 is assumed) indicates acute organ dysfunction, and the diagnosis of sepsis is met if an infection is identified in parallel. If, in addition, hypotension is seen (that is, mean blood pressure of at least 65 mmHg can be reached only using vasopressors, despite adequate fluid management) and the serum lactate levels are more than 2 mmol/L, one speaks of “septic shock”. Sepsis-3 also provides a new tool meant as a simplified screening tool for early recognition of organ dysfunction because of infection: qSOFA (“quickSOFA”) ^15^. It is intended primarily for use in emergency departments, peripheral wards, rest homes, and so on and not in ICUs, and it consists of the following three criteria:

• altered mental status
• respiratory rate of more than 22 breaths/minute
• systolic blood pressure of less than 100 mmHg.

When two of these three qSOFA criteria are met, organ dysfunction should be suspected, and the classic SOFA score should be determined by experienced physicians, usually intensive care specialists.

## Sepsis without “SIRS”: is it feasible?

In 2015, an Australian study was published that used a large database to determine the influence of SIRS on prognosis ^19^. The results may be summarized as follows: first, the presence of SIRS (defined by two or more SIRS criteria) did not influence the overall prognosis; second, about every eighth patient is missed if SIRS is necessary for defining sepsis; third, even though an increasing trend in mortality was seen according to increasing numbers of observed SIRS criteria, no significant difference in mortality rate could be identified, especially comparing patients with zero versus one or one versus two SIRS criteria ^19^. These results were confirmed by the Sepsis-3 task force; the basis for this consisted of large data sets of hospitalized patients with suspected infection that were used to assess the validity of several diagnostic and critical care scores. Core results were the low specificity of SIRS (whether in critical care or on peripheral wards), the high prognostic value of the SOFA score in ICUs, and the high prognostic value of a change in cognitive status, respiratory rate, and systolic pressure (qSOFA), particularly in non-ICU patients ^16^.

In conclusion, the weakness of the old, SIRS-based Sepsis-1 definition is obvious and was demonstrated by high-level scientific research ^19^. Hence, we actually do need a new definition and should no longer use the “SIRS yes or no” criterion for defining sepsis. The new Sepsis-3 definition is based on sound and extended statistical analyses, thus providing a good basis for use in future clinical research ^15– 17^. Furthermore, qSOFA was demonstrated to be a useful tool but needs further validation studies. Finally, the “SIRS” criteria (put in quotation marks, since the authors think that the former entity SIRS should no longer be used) leukocytes, heart rate, and temperature are still important to detect infections but should no longer be used to diagnose sepsis. These two sides of the coin—qSOFA as a screening tool in suspected organ dysfunction plus leukocytes, heart rate, and temperature for surveillance in patients endangered by infections—should be established as a clinical quality standard.

## Quality improvement in sepsis management

Around 15 years ago, three international research societies founded the SSC, aiming to reduce sepsis mortality by more than 25% (relative risk reduction) over 5 years. One tool was the creation of international guidelines for the management of sepsis; the first version was published in 2004 and the third revision in 2017 ^20^. Based on the first version, “SSC Sepsis Bundles” were created, consisting of several measurable interventions (for example, antibiotics, blood cultures, and serum lactate measurement). According to the initial plan, these bundles were tested in more than 30,000 cases of sepsis and septic shock worldwide over a period of 5 years (2005–2010). As a result, it was demonstrated that (1) the planned mortality reduction was reached and (2) not all parts of the bundles had an intrinsic effect on patient outcome ^21^.

In parallel, several comparable projects were started all over the world—for example, in Spain (“Edusepsis Group”)—which led to similar results ^22^. In 2014, a follow-up study over 7.5 years from the SSC Bundle Project confirmed the data from 2010, and the authors concluded that “These results demonstrate that performance metrics can drive change in clinical behavior, improve quality of care, and may decrease mortality in patients with severe sepsis and septic shock” ^23^. Recently, a German group published data, again from a 7.5-year period using a hospital-supported quality improvement program to reduce sepsis mortality ^24^. In more than 14,000 included patients, 90-day mortality decreased significantly, from 64.2% to 45.0%, and the length of stay in the hospital decreased from 44 to 36 days.

## Recent development in sepsis management

The aforementioned improvement in survival of patients with sepsis and septic shock by standardized protocols and related control instruments (“standard operating procedures”, “check lists”, and so on) led to a broad discussion of whether these protocols should be a mandatory quality indicator. Based on private activities from a New York state (USA) family that was affected by a lethal case of sepsis, the New York State Department of Health in 2013 decided that all state hospitals have to implement evidence-informed protocols for the fast management of sepsis and septic shock (New York Codes, Rules, and Regulations parts 405.2 and 405.4). The way this was carried out could be decided by the hospitals themselves, but the minimum requirement was a 3-hour bundle with the following interventions: (1) blood cultures before administration of antibiotics, (2) serum lactate measurement, and (3) infusion of broad-spectrum antibiotics. Although in the treatment of patients with sepsis there are many more options that provided a beneficial effect (for example, protective mechanical ventilation with low tidal volumes), these regulations concentrated on early treatment, within hours after the detection of sepsis, and therefore included mainly emergency departments.

In 2017, the results from the first 2.25 years after starting these rules were published, presenting data from 149 hospitals, including more than 49,000 patients ^25^; 82.5% of these met the criteria for the 3-hour bundle. Furthermore, it was demonstrated that each 1-hour delay—measured from the initial time of detecting sepsis—increased mortality by 4% (relative risk). Similar results were found for the single interventions of blood culture, antibiotics, and lactate measurement, whereas the effect of early fluid administration was demonstrated only in septic shock patients with a need for vasopressor administration ^25^. This latter point supports current discussions that an early fluid challenge might not be favorable in every patient with sepsis (so-called “fluid non-responder”) and that fluid administration should be monitored carefully to avoid a fluid overload with negative effects on patient outcome.

In regard to the effect of early antibiotics, these data did not support the concern that there might be a risk of increasing antibiotic resistance as pointed out by Singer ^26^. Furthermore, a recent article by Ferrer *et al*. demonstrated that an improvement of a more rapid microbiological diagnosis in parallel with early antibiotics facilitates selection of antibiotics and improves outcome ^27^. In contrast, new data show that for the source control of infection, the identification of the location, rather than time, might be the most important parameter of improved outcome. Martínez *et al*. revealed data that outcome may vary according to the source of infection and that urinary tract infection with subsequent sepsis is associated with a lower mortality compared with severe pneumonia ^28^. Finally, the microorganism’s virulence and bacterial load may influence the prognosis of sepsis ^29^.

Based on these impressive data, the SSC steering group recently published newly defined “SSC Sepsis Bundles 2018”, which are now based on a 1-hour period ^30^:

• Measure lactate level. Re-measure if the initial level is more than 2 mmol/L.
• Obtain blood cultures prior to the administration of antibiotics.
• Administer broad-spectrum antibiotics.
• Begin rapid administration of 30 mL/kg crystalloid for hypotension or lactate of at least 4 mmol/L.
• Apply vasopressors if the patient is hypotensive during or after fluid administration to maintain mean arterial pressure of at least 65 mmHg.

“Time zero” or “time of presentation” is defined as the time of triage in the emergency department or, if presenting from another care venue, from the earliest chart annotation consistent with all elements of sepsis (formerly severe sepsis) or septic shock ascertained through chart review.

Since these new bundles were published very recently, no validation studies have been performed so far. However, the discussion started within days; many clinicians are very critical of this “mandatory approach”. Two major points (but not the only ones) are that (1) it is presumed that hospitals will perform these bundles more or less “in any case” of sepsis suspicion, even if there may be some time to wait for a more thorough diagnosis, since they fear reduced reimbursement or even legal action, and that (2) the possible “over-therapy”, especially with early administration of antibiotics, may induce side effects for the treated patients as well as a higher rate of resistance against antibiotics over time.

## Closing remarks

In patients with sepsis or septic shock, a better understanding of the host response leading to the clinical course, a faster detection of high-risk patients, and an earlier and more standardized approach in managing sepsis are the key challenges in current clinical practice. On the one hand, the recent findings of host defense mechanisms on the cellular level, the new Sepsis-3 definition, and the current developments after investigating the effects of mandatory care of patients with sepsis are significant and promising steps in sepsis research. On the other hand, these steps are tracking new ways which—in some cases—may lead to unknown destinations. At present, it is too early to risk a clear prognosis; we all hope that the sum effect of these new developments will be a positive one. At least it was demonstrated that advances in sepsis research are possible! Perhaps this will foster research engagement by clinicians and scientists in this exciting field of medicine and will bring more attention and support from industrial as well as public institutions.
